# Supplementary material for: Novel features of centriole polarity and cartwheel stacking revealed by cryo‐tomography
Source: EMBO J. 2020 Sep 20;39(22):e106249. doi: 10.15252/embj.2020106249 (PMC7667878; doi:10.15252/embj.2020106249)
Supplement: Supplementary file 1 — Appendix [file EMBJ-39-e106249-s001.pdf]

Appendix

Novel features of centriole polarity and cartwheel stacking revealed by cryo-tomography

Sergey Nazarov, Alexandra Bezler, Georgios N Hatzopoulos, Veronika Nemčíková Villímová, Davide Demurtas, Maeva Le Guennec, Paul Guichard and Pierre Gönczy

Correspondence: pierre.gonczy@epfl.ch

Table of Contents

Appendix Figure S1 - pages 2-3

Appendix Table S1 - page 4

Appendix Figure S2 - pages 5-6

Appendix Figure S3 - pages 6-7

Appendix Figure S4 - page 8-9

Appendix Figure S5 - page 9

Appendix Table S2 - page 10

 © 2020 The Authors

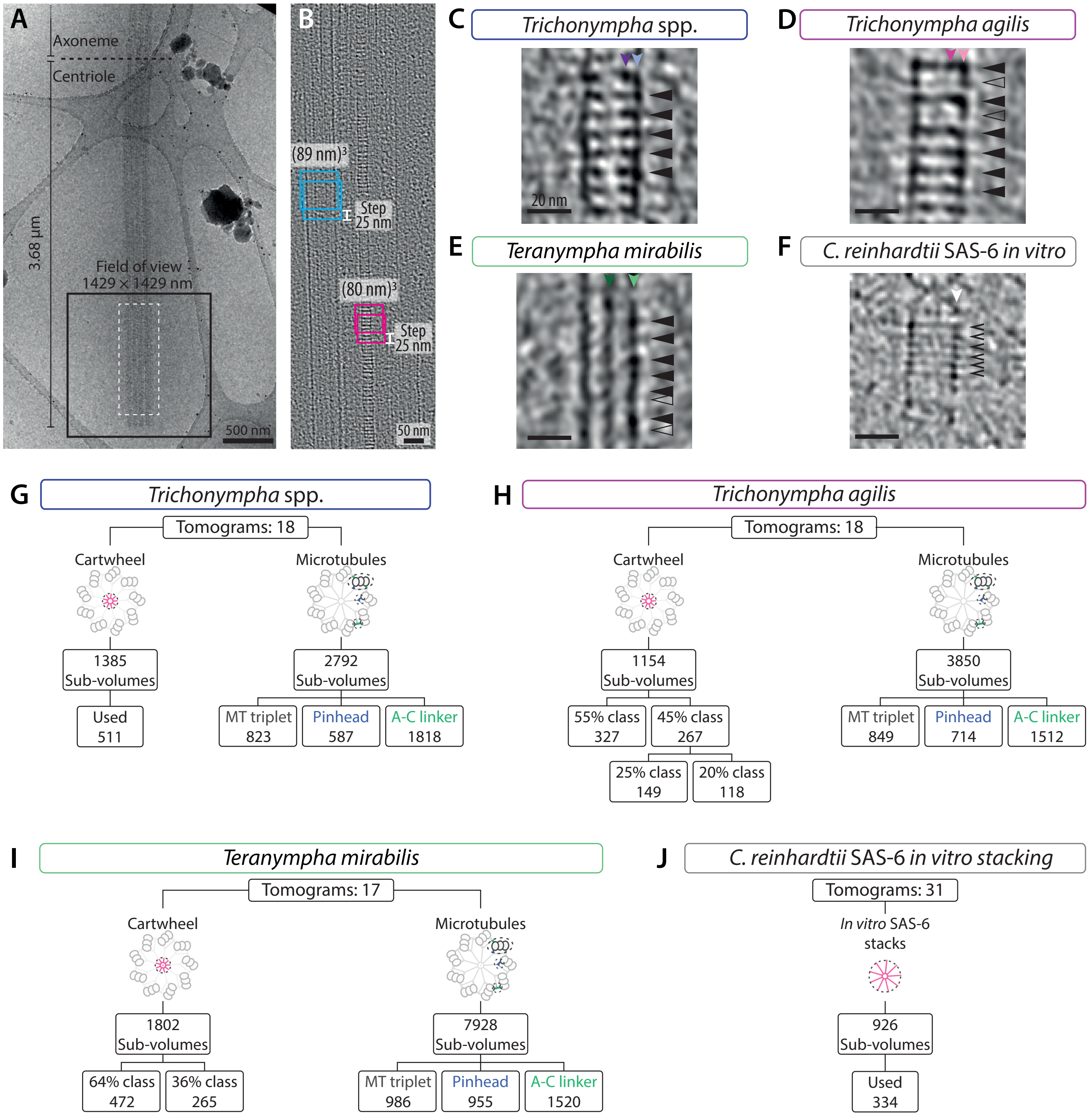

Appendix Figure S1

© 2020 The Authors

Appendix Figure S1: Cryo-EM acquisition, raw data and processing

A Low magnification view of cryo-preserved purified *T.* spp. centriole and axoneme on carbon grid. Note that the proximal end of the centriole is blunt (bottom), and that the diameter of the centriole containing microtubule triplets is larger than that of the axoneme containing microtubule doublets (top), enabling one to determine the length of the centriole, as indicated. The black square illustrates the proximal location and the 1429 x 1429 nm size of the field of view typically used for tilt series acquisition; note that this can be less than the length of the centriole depending on the species. Dashed white rectangle: area corresponding to the tomogram shown in (B).

B 2D longitudinal view (sum of 20 slices) of the central cartwheel tomogram, with band-pass filter and Gaussian blur corresponding to the area with a dashed rectangle in (A). Sub-volumes extracted along the longitudinal axis are $(80\ nm)^3$ for the central cartwheel (pink squares represent one plane of the sub-volume) and $(89\ nm)^3$ for the peripheral elements comprising microtubule triplet, pinhead and A-C linker (blue squares represent one plane of the sub-volume), with a step size of 25 nm in both cases.

C-F 2D longitudinal views from raw reconstructed tomograms of the central cartwheel illustrating typical sub-volumes (sum of 10-20 slices of the central cartwheel tomogram, with band-pass filter and Gaussian blur) from *T.* spp. (C), *T. agilis* (D), *T. mirabilis* (E) and CrSAS-6[NL] stacked assemblies (F). Colored arrowheads point to CID/fCID and hub positions; colored as in Fig. 2 and Fig. 4. Filled arrowheads indicate hub elements constituted of double units that can sometimes be resolved upon STA, but are not visible in this raw data (C-E); empty arrowheads indicate hub elements in the *T. agilis* 45 % class lacking the CID (D). Note that classes cannot be unambiguously distinguished in the *T. mirabilis* raw tomograms; empty arrowheads in this case indicate hub elements with apparent variations in spacing (E). Chevrons indicate resolved CrSAS-6[NL] single unit hub elements (F).

G-J Processing scheme with number of tomograms, initial sub-volumes extracted for cartwheel and peripheral elements (microtubule triplet, pinhead and A-C linker), classification/refinement steps, and final sub-volumes used for STA in *T.* spp. (G), *T. agilis* (H), *T. mirabilis* (I) and CrSAS-6[NL] stacked assemblies (J). See also Appendix Table S1.

 © 2020 The Authors

sample	*T. spp.*	*T. agilis*	*T. mirabilis*	*C. reinhardtii*
	centrioles			*in vitro* CrSAS-6
**Data collection**				
TEM	F20			Krios
Pixel size (Å)	3.49			2.71
Tilting scheme	Bi-directional			Dose-symmetric
N tomograms	18	18	17	31
**Reconstruction: cartwheel**				
Step size for extraction (Å)	3 x 85 = 255			2 x 42 = 84
extracted sub-volumes	1385	1154	1802	926
sub-volumes final	511	594	737	334
Resolution CW (Å)	24	23	23	15
EMDB accession	*EMD-10927*	*EMD-10916*<br>*EMD-10918*	*EMD-10922*<br>*EMD-10923*	*EMD-10928*
**Reconstruction: microtubule triplet (MTT), pinhead (PH), A-C linker (AC)**				
Step size for extraction (Å)	3 x 85 = 255			na
extracted sub-volumes	2792	3850	7928	na
sub-volumes MTT, final	823	849	986	na
Resolution MTT (Å)	38	40	38	na
EMDB accession	*EMD-10932*	*EMD-10931*	*EMD-10934*	na
sub-volumes PH, final	587	714	955	na
Resolution PH (Å)	39	39	37	na
EMDB accession	*EMD-10938*	*EMD-10935*	*EMD-10941*	na
sub-volumes AC, final	1818	1512	1520	na
Resolution AC (Å)	34	37	37	na
EMDB accession	*EMD-10939*	*EMD-10937*	*EMD-10942*	na

Appendix Table S1: Statistics of Cryo-EM data collection, reconstruction, and EMDB accession codes; see also Appendix Fig S1

 © 2020 The Authors

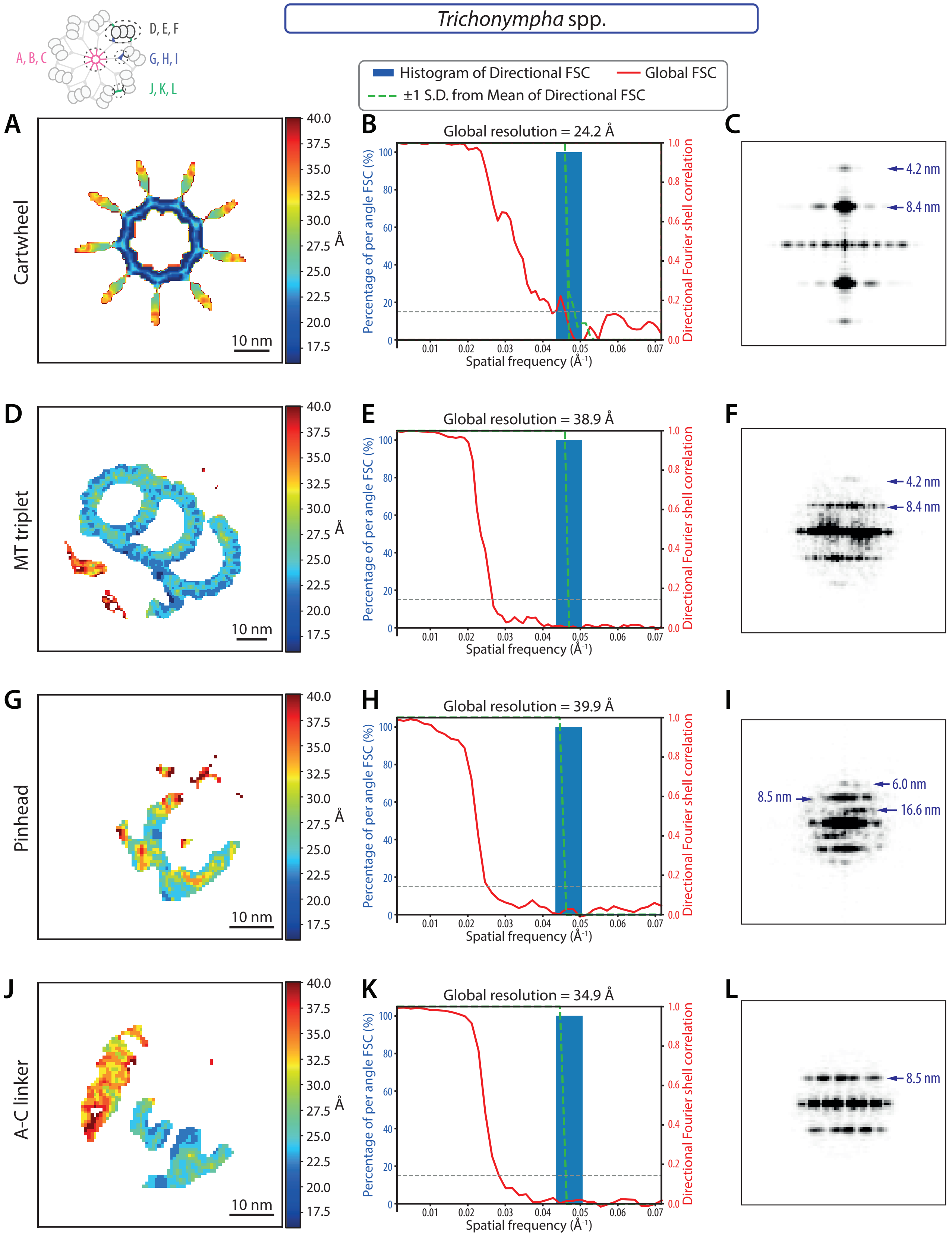

Appendix Figure S2

 © 2020 The Authors

Appendix Figure S2: Resolution maps of *T.* spp.

A-C *T.* spp. central cartwheel STA local resolution distribution estimated with ResMap in this and analogous panels (A), histogram and directional Fourier shell correlation (FSC) plots with global resolution at FSC 0.143 criterion in this and analogous panels (B), as well as power spectrum of 2D class average with periodicities highlighted by arrows (C). The measurement precision in this and remaining power spectra is 1 pixel, corresponding to a minimum of 0.4 nm (see Appendix Table S1). Schematic on top left illustrates the different areas used to generate maps of the central cartwheel, microtubule triplets, pinhead and A-C linker.

B-F *T.* spp. microtubule triplet STA local resolution distribution (D), histogram and directional FSC plots (E), and power spectrum of 2D class average with periodicities highlighted by arrows (F).

G-I *T.* spp. pinhead STA local resolution distribution (G), histogram and directional FSC plots (H), and power spectrum of 2D class average with periodicities highlighted by arrows (I).

J-L *T.* spp. A-C linker STA local resolution distribution (J), histogram and directional FSC plots (K), and power spectrum of 2D class average with periodicity highlighted by arrows (L).

Appendix Figure S3: Resolution maps of *T. agilis*

A-C *T. agilis* central cartwheel STA 55 % class (A-C) and 45 % class (D-F); local resolution distribution (A and D), histogram and directional FSC plots (B and E), and power spectra of 2D class average with periodicities highlighted by arrows (C and F). Schematic on top left illustrates the different areas used to generate maps of the central cartwheel, microtubule triplets, pinhead and A-C linker.

G-I *T. agilis* microtubule triplet STA local resolution distribution (G), histogram and FSC plots (H), and power spectrum of 2D class average with periodicity highlighted (I).

J-L *T. agilis* pinhead STA local resolution distribution (J), histogram and directional FSC plots (K), and power spectrum of 2D class average with periodicities highlighted (L).

M-O *T. agilis* A-C linker STA local resolution distribution (M), histogram and FSC plots (N), and power spectrum of 2D class average with periodicity highlighted (O).

© 2020 The Authors

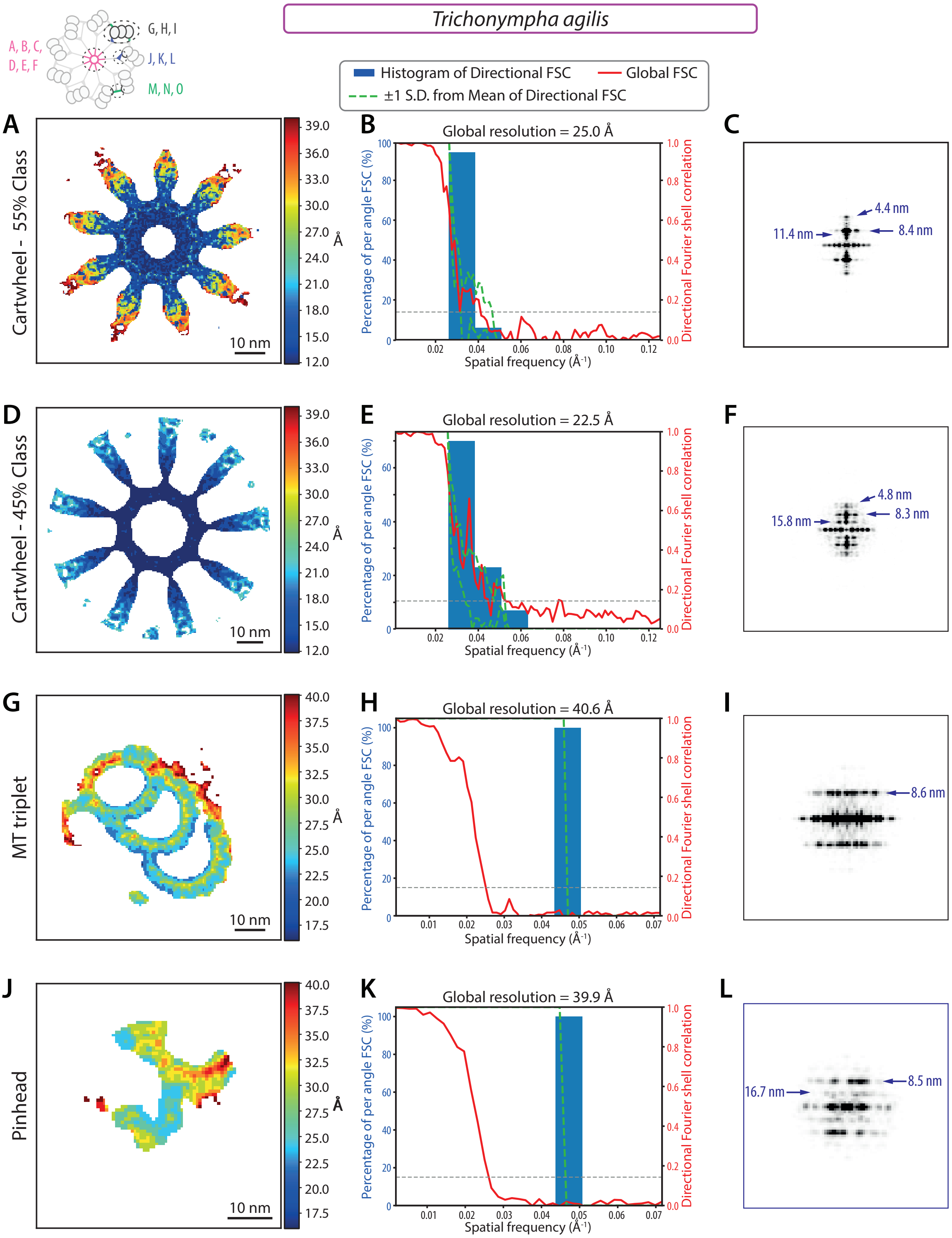

Appendix Figure S3

© 2020 The Authors

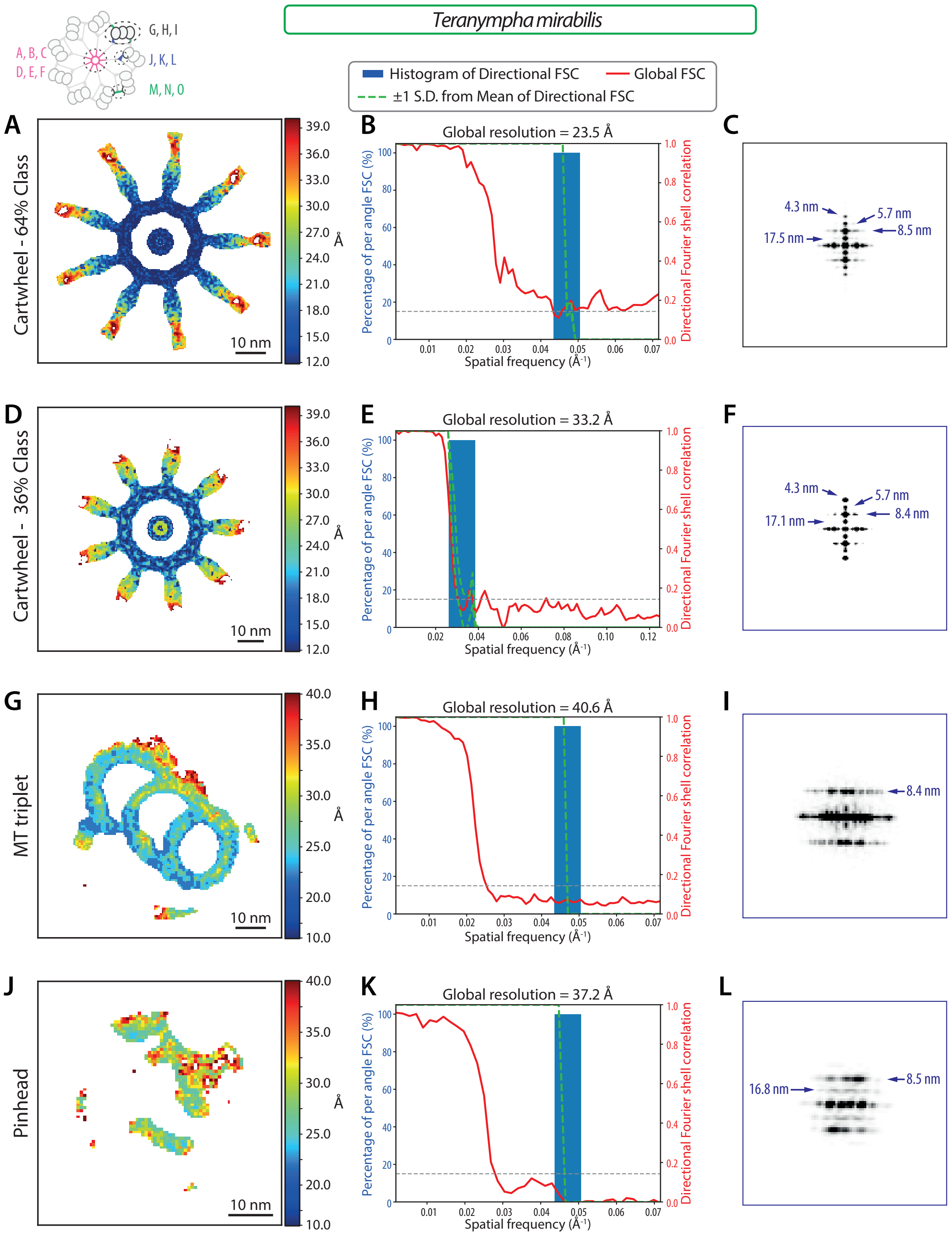

Appendix Figure S4

© 2020 The Authors

Appendix Figure S4: Resolution maps of *T. mirabilis*

A-F *T. mirabilis* central cartwheel STA 64 % class (A-C) and 36 % class (D-F), local resolution distribution (A and D), histogram and directional FSC plots (B and E), and power spectra of 2D class average with periodicities highlighted by arrows (C and F). Schematic on top left illustrates the different areas used to generate maps of the central cartwheel, microtubule triplets, pinhead and A-C linker.

G-I *T. mirabilis* microtubule triplet STA local resolution distribution (G), histogram and FSC plots (H), and power spectrum of 2D class average with periodicities highlighted (I).

J-L *T. mirabilis* pinhead STA local resolution distribution (J), histogram and directional FSC plots (K), and power spectrum of 2D class average with periodicities highlighted (L).

M-O *T. mirabilis* A-C linker STA local resolution distribution (M), histogram and FSC plots (N), and power spectrum of 2D class average with periodicity highlighted (O).

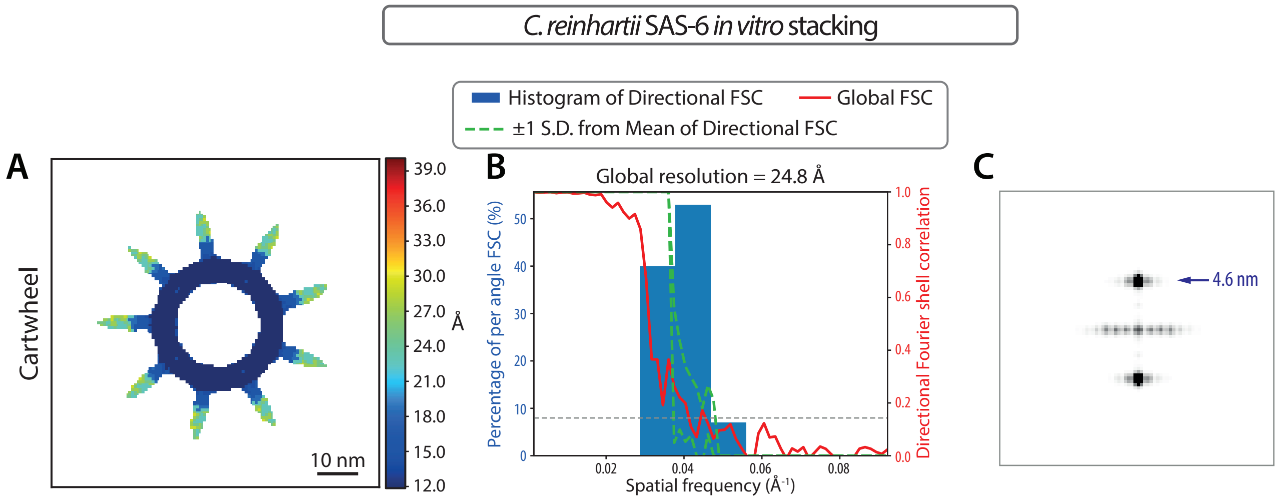

Appendix Figure S5: Resolution map of CrSAS-6[NL] stacked assemblies

A-C CrSAS-6[NL] stacked assemblies STA (generated from side-views) local resolution distribution shown in transverse view (A), histogram and directional FSC plots (B), and power spectrum of 2D class average with periodicity highlighted by arrow (C).

 © 2020 The Authors

Map	Ring model	Percentage (%) of atoms inside contour	Correlation	Overlap	Percentage (%) gain in overlap score by fitting double over single ring
T. mirabilis 36% class	Single	77	0.83	23	
	Double	63	0.71	31	+58
	Offset	69	0.73	27	+87
T. mirabilis 64% class	Single	93	0.78	17	
	Double	78	0.71	26	+47
	Offset	71	0.74	34	+47
***T.* spp.**	Single	94	0.83	34	
	Double	64	0.65	41	+23
	Offset	63	0.69	45	+33
T. agilis 55% class	Single	99	0.86	38	
	Double	82	0.73	51	+34
	Offset	86	0.78	55	+44
T. agilis 45% class - thick	Single	100	0.86	41	
	Double	87	0.79	60	+46
	Offset	78	0.83	64	+56
T. agilis 45% class - thin	Single	93	0.87	33	
	Double	46	0.63	34	+4
	Offset	46	0.63	34	+4
**CrSAS-6[NL]** *in vitro*	Single	91	0.90	18	
	Double	91	0.90	35	+97
	Offset	84	0.88	33	+83

Appendix Table S2: Correlation and overlap score for fitting with computationally assembled CrSAS-6[6HR] single, double, and double offset rings

Results of the fit in map function performed with ChimeraX (Goddard *et al*, 2018) by maximizing correlation as shown in Fig 5, Fig EV4, Fig EV5 and described in Materials and Methods. Correlation and overlap are calculated with ChimeraX; correlation value range: -1 to 1. Reported are the percentage of atoms inside the density map, the correlation, and the overlap between model and map. For each STA map, we report the gain in overlap upon fitting the double and offset double versus single ring model; the maximum possible gain is +100%.

© 2020 The Authors
