# Supplementary material for: Novel features of centriole polarity and cartwheel stacking revealed by cryo‐tomography
Source: EMBO J. 2020 Sep 20;39(22):e106249. doi: 10.15252/embj.2020106249 (PMC7667878; doi:10.15252/embj.2020106249)
Supplement: Supplementary file 2 — Expanded View Figures PDF [file EMBJ-39-e106249-s002.pdf]

Expanded View Figures

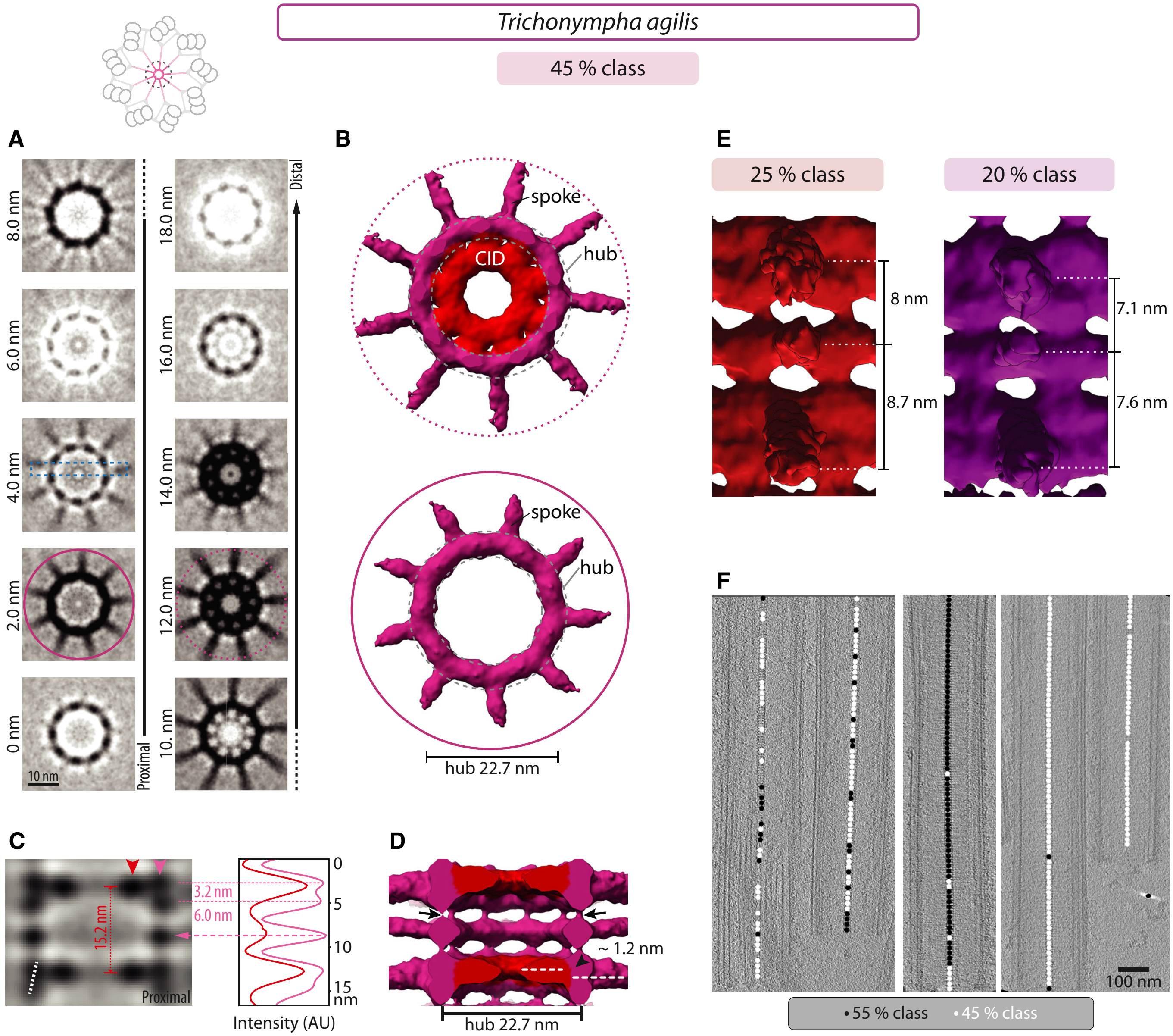

Figure EV1.

 © 2020 The Authors

Figure EV1. Variation in hub architecture in some *T. agilis* sub-volumes.

A Transverse 2D slices through central cartwheel STA of *T. agilis* 45% class, which comprises 25 and 20% sub-classes (see E), at indicated height from proximal (0 nm) to distal (18.0 nm). The pink circles mark spokes with CID (12.0 nm, dashed line) or without CID (2.0 nm, solid line), as represented in (B); dashed box in the 4.0 nm slice indicates longitudinal section shown in (C). Schematic on top illustrates the area used to generate the 3D maps of the central cartwheel.

B Transverse views of central cartwheel STA 3D map of *T. agilis* 45% class. The hub diameter is 22.7 nm $\pm$ 0.2 nm (N = 3) with 9 emanating spoke densities, either at a level where the CID is present (dashed circle) or absent (solid circle), as indicated in (A).

C 2D longitudinal view of central cartwheel STA of *T. agilis* 45% class delineated by a dashed box in (A). Arrowheads denote position of line scans along the vertical axis at the level of the CID (red) and the hub (pink), with corresponding normalized pixel intensities in arbitrary units (AU). The plot profiles are shifted horizontally relative to each other for better visibility. The distance between hub densities alternates between 3.2 nm (N = 1) and 6.0 $\pm$ 0.3 nm (N = 2); maxima are indicated by dashed pink lines. Dashed white line indicates offset of two superimposed hub units. The average distance between two CID elements is 15.2 nm (N = 1; dashed red line). The middle hub density that comprises only one unit and lacks a neighboring CID is indicated by a dashed arrow.

D Longitudinal view of central cartwheel STA of *T. agilis* 45% class at lower contour level than in (B). Note densities bridging successive hubs vertically (arrows), as well as proximal location of CID relative to the spoke density axis, resulting in a vertical offset of 1.2 $\pm$ 0 nm (N = 2; arrowhead). Note also absence of CID in middle hub element comprising a single unit.

E The spacing between the spoke density emanating from a double hub unit and the spoke density emanating from a single hub unit distal to it is 8.0 nm in the 25% class and 7.1 nm in the 20% class (both N = 1). By contrast, the spacing between the spoke density emanating from a double hub unit and the spoke density emanating from a single hub unit proximal to it is 8.7 nm in the 25% class and 7.6 nm in the 20% class (both N = 1).

F Distribution of sub-volumes of the 55% (black circle) and 45% (white circle) classes along five *T. agilis* centrioles; areas with neither black nor white circle could not be clearly assigned to either class. Note individual centrioles constituted of mostly one of the two classes, and others where the two classes are mixed without an apparent pattern, indicating that the distribution of 55% and 45% classes is not stereotyped along the centriole.

Figure EV2. Polar centriolar cartwheel.

A–P Non-symmetrized STA comprising larger sub-volumes than in Fig 2-4 and centered on the spokes to jointly show the central cartwheel and peripheral elements in *T. agilis* (A-D: 55% class; E-H: 45% class) and *T. mirabilis* (I-L: 64% class; M-P: 36% class). 2D slices through STA transverse view (A, E, I, M) with corresponding 3D views (B, F, J, N), as well as 2D longitudinal views (C, G, K, O) with corresponding 3D views (D, H, L, P). The concerted proximal–distal polarity is visible from the central CID (in A-H) and the hub all the way to the pinhead. Note that proximal–distal polarity is visible also in the asymmetric spoke tilt angles in all cases, with a more pronounced tilt on the proximal side (D, H, L, P). Note also that the unsymmetrized CID shown here resembles that in the symmetrized maps of Figure 2, indicating that the CID exhibits *bona fide* 9-fold radial symmetry (A, E). Arrowhead in (N, P) points to a connection between fCID and hub. For representation, a Gaussian filter was applied to maps in ChimeraX.

© 2020 The Authors

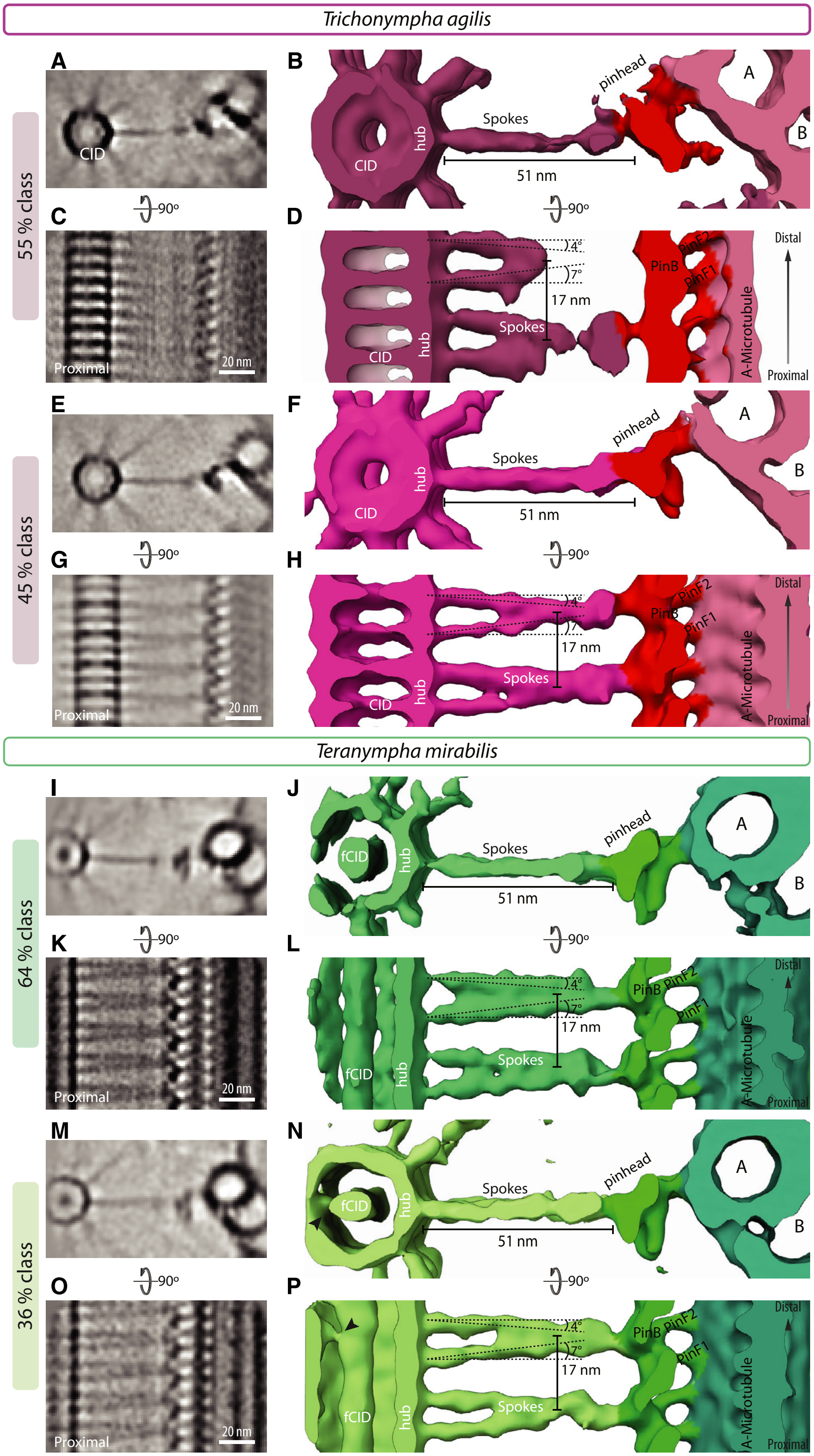

Figure EV2.

 © 2020 The Authors

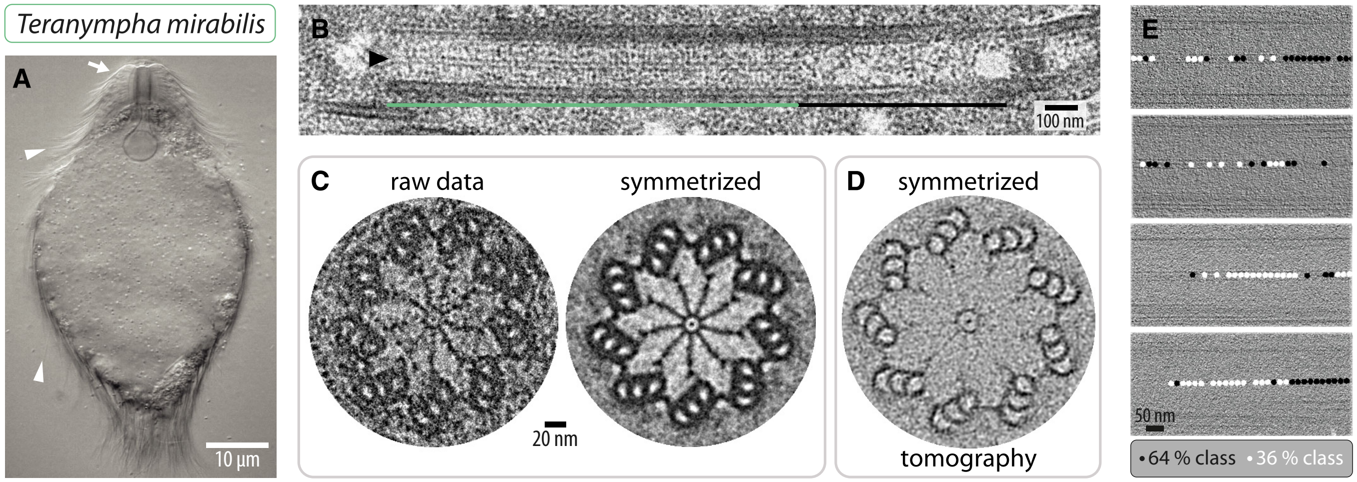

Figure EV3. Very long cartwheel in *T. mirabilis*.

A Differential interference contrast micrograph of live *T. mirabilis* cell. The arrow points to the cell anterior, where the rostrum is located; arrowheads point to some of the flagella.

B Transmission electron micrograph of *T. mirabilis* centriole embedded in resin—longitudinal view. The hub (arrowhead) is visible in the proximal cartwheel-bearing region (green line), but not in the distal region (black line).

C Transmission electron micrograph of *T. mirabilis* centriole embedded in resin in transverse view (left) and corresponding image circularized and symmetrized (right). Note small density present inside the hub corresponding to the fCID. Centrioles shrink during chemical fixation and subsequent preparation, thus appearing smaller than in (D).

D Transverse slice through cryo-electron tomogram of *T. mirabilis* centriole circularized and symmetrized. Note the fCID presence inside the hub.

E Distribution of sub-volumes of the 64% (black circle) and 36% (white circle) classes along four *T. mirabilis* centrioles, proximal is left; areas with neither black nor white circle could not be clearly assigned to either class. Note that the distribution of the two classes is not stereotyped along the centriole.

© 2020 The Authors

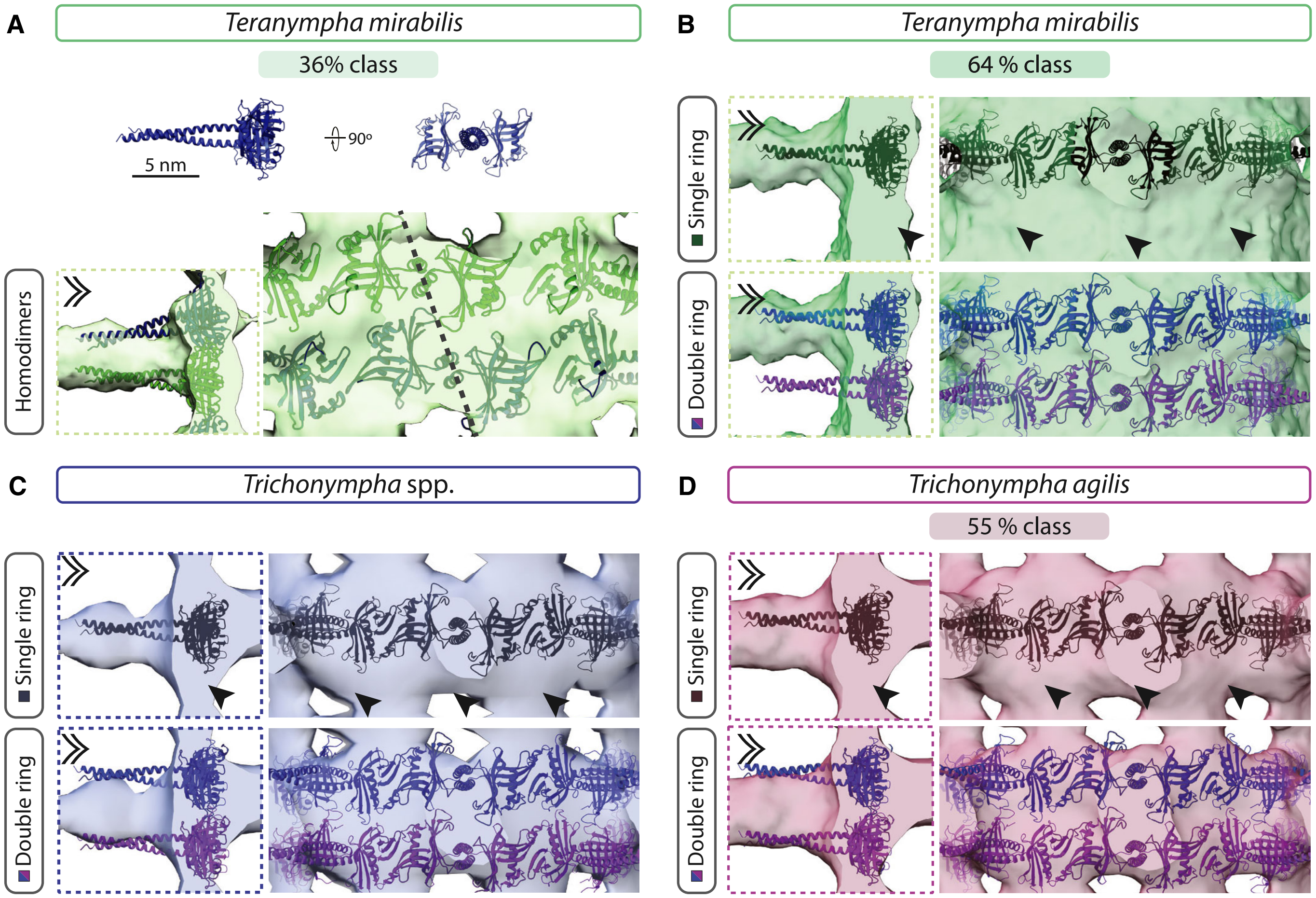

Figure EV4. CrSAS-6[6HR] fitting in different cartwheel STA maps.

A Ribbon representation of CrSAS-6[6HR] homodimers (top) shown magnified in side view (left) and end-on view (right) that were used for rigid-body fitting into the 3D map from *T. mirabilis* 36% class (bottom), with dashed line indicating offset between two superimposed homodimers. Individual layers of homodimers are indicated in distinct colors for clarity. In this and all other panels of this figure, dashed box indicates longitudinal section through hub element (left), and double chevron indicates viewing direction of longitudinal external views (right).

B–D CrSAS-6[6HR] single or double rings in register (ribbon diagram represented in different shades for clarity) fitted into the 3D maps from the *T. mirabilis* 64% class (B), *T.* spp. (C) and the *T. agilis* 55% class (D). Single ring fitting is shown on top, double ring fitting at the bottom. Note unaccounted densities in the hub upon fitting of single rings in all cryo-ET maps (arrowheads, B-D), whereas all maps readily accommodate double CrSAS-6[6HR] rings at the hub element, although the coiled-coil elements extend slightly beyond the spoke densities. Note also that one coiled-coil in the *T. mirabilis* 64% class sticks out of the density entirely (B, bottom).

© 2020 The Authors

Figure EV5. Superimposed rings of SAS-6 may be offset with respect to one another.

A, B (Left) Ribbon representation of CrSAS-6[6HR] homodimers computationally assembled into double rings (rings represented in different colors for clarity) with spokes in register (A) or with a 6.5° offset turning the proximal ring clockwise and moved 0.4 nm closer to one another (B); transverse (top) and longitudinal view (bottom). (Right top) Magnified views of two vertically superimposed homodimers assembled in a ring (ribbon diagram), with dashed lines indicating the offset angle between the two coiled-coil axes. (Right bottom) Surface representation of interface; note the increased complementarity in (B). Dashed lines indicate offset between rings. Distances were measured on the ribbon diagram.

C, D CrSAS-6[6HR] offset double rings (shown in B and represented in different colors for clarity) fitted into 3D map from *T. mirabilis* 36% class (C) and *T. agilis* 45% class (D), with dashed line indicating offset between rings. Dashed box indicates longitudinal section through hub element (left), double chevron viewing point of longitudinal external views (right).

E Schematic illustrating processing reported in (F-H): After linearization of the cartwheel STA, a longitudinal section in the Z-direction (blue line) is shown at the level of the spokes (represented in pink).

F–H Longitudinal section as explained in (E) for *T. mirabilis* 36 and 64% classes (F), *T. agilis* 45 and 55% classes (G) and *T.* spp. (H) at the level of spoke emergence from the hub. Note offset visible by the elliptic shape of individual spokes and highlighted by a dashed line.

I Longitudinal section as explained in (E) for *in vitro* assembled CrSAS-6[NL] stacks; spokes from consecutive stacks are almost in register. Note that in (F-H) the spokes of double hub elements cannot be fully resolved, whereas they appear as individual units in (I).

© 2020 The Authors

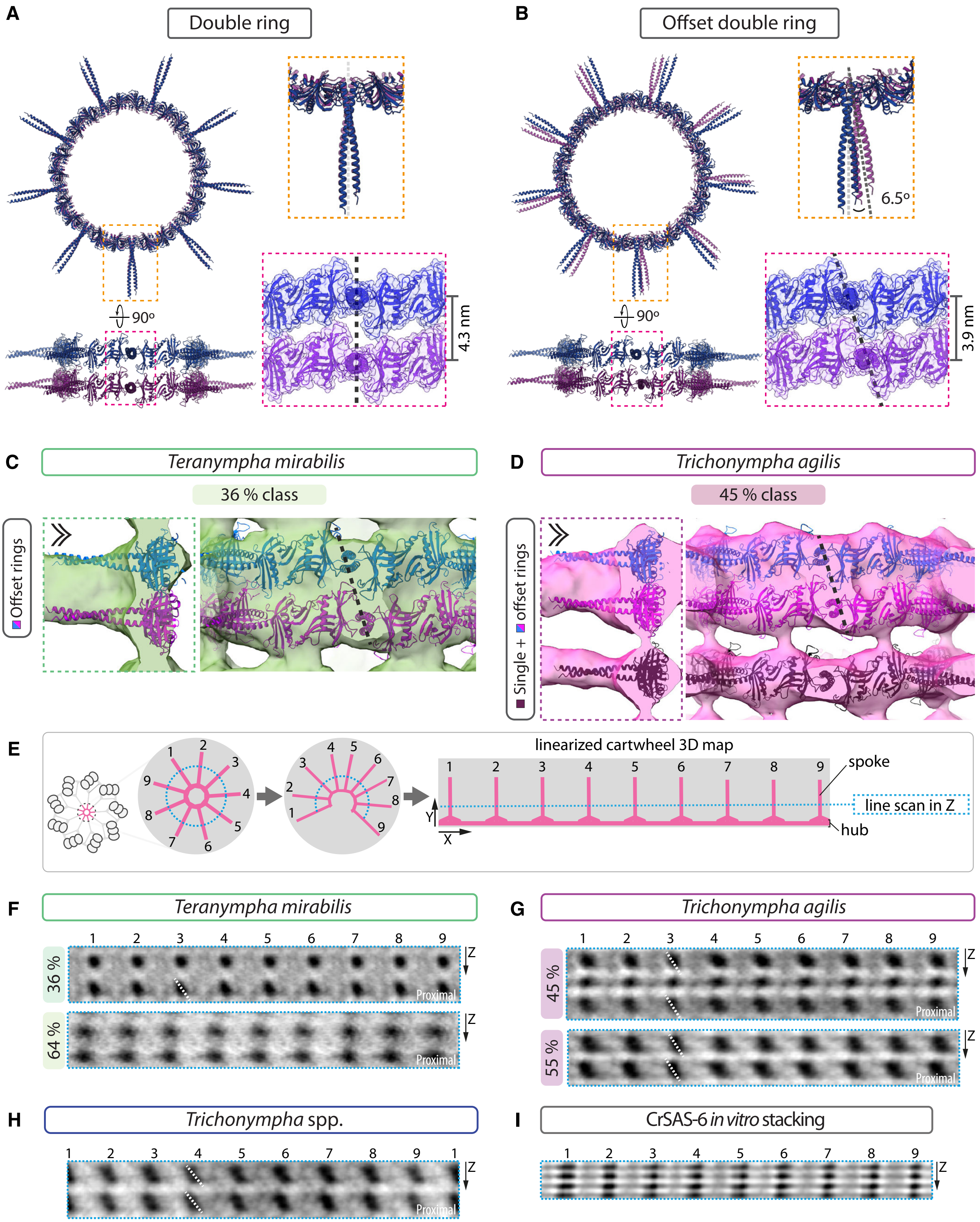

Figure EV5.

 © 2020 The Authors
